# Role of raising the upper limb of the non-rising side when performing rising movements from bed

**DOI:** 10.1038/s41598-023-38779-2

**Published:** 2023-07-16

**Authors:** K. Hirata, H. Hanawa, T. Miyazawa, K. Kubota, M. Yokoyama

**Affiliations:** 1 Department of Rehabilitation, Faculty of Health Sciences, Tokyo Kasei University, 2-15-1 Inariyama, Sayama, Saitama 350-1398 Japan; 2 Graduate Course of Health and Social Services, Graduate School of Saitama Prefectural University, Saitama, Japan; 3 Department of Rehabilitation, Faculty of Health Science, University of Human Arts and Sciences, Saitama, Japan; 4 Research Development Center, Saitama Prefectural University, Saitama, Japan; 5 Sportology Center, Graduate School of Medicine, Juntendo University, Tokyo, Japan

**Keywords:** Rehabilitation, Geriatrics, Musculoskeletal system

## Abstract

Rising movements from bed comprise an important aspect of recovery from the bedridden state; however, they have not been sufficiently investigated using motion analysis studies. In particular, the effect of using the upper limb of the non-rising side before waist flexion on rising movements remains to be analyzed; this study aimed to clarify this effect. Accordingly, motion analyses were performed on rising movements under two constraint conditions, namely raising the upper limb of the non-rising side (upper limb use-condition) and keeping it in contact with the pelvis (upper limb non-use-condition); subsequently, the kinematics and kinematics parameters were compared. In comparison with the upper limb use-condition, in the upper limb non-use-condition, the distance traveled by the center of mass of the body (CoM trajectory, p < 0.01) increased while switching from the half-side-lying to on-hand postures, horizontal body movement (movement speed (Normalized time/total time), p < 0.01 and weight of center of body mass (CoM momentum in horizontal plane), p < 0.05) during the same period increased, and the half-side-lying time approached the peak value of the waist flexion angular velocity (Time lag between from half-side-lying to waist angler peak velocity, p < 0.05). The compensatory movement that occurred due to the upper limb non-use-condition denoted an increase in body momentum in the horizontal direction, rather than in the sagittal plane. Therefore, the upper limb on the non-rising side contributed to the smooth movement of the body in the horizontal direction. Moreover, this study demonstrated that asymmetrical rising movement in the diagonal direction is a characteristic movement wherein the horizontal movement of the body constitutes the main movement.

## Introduction

**Figure 1:**
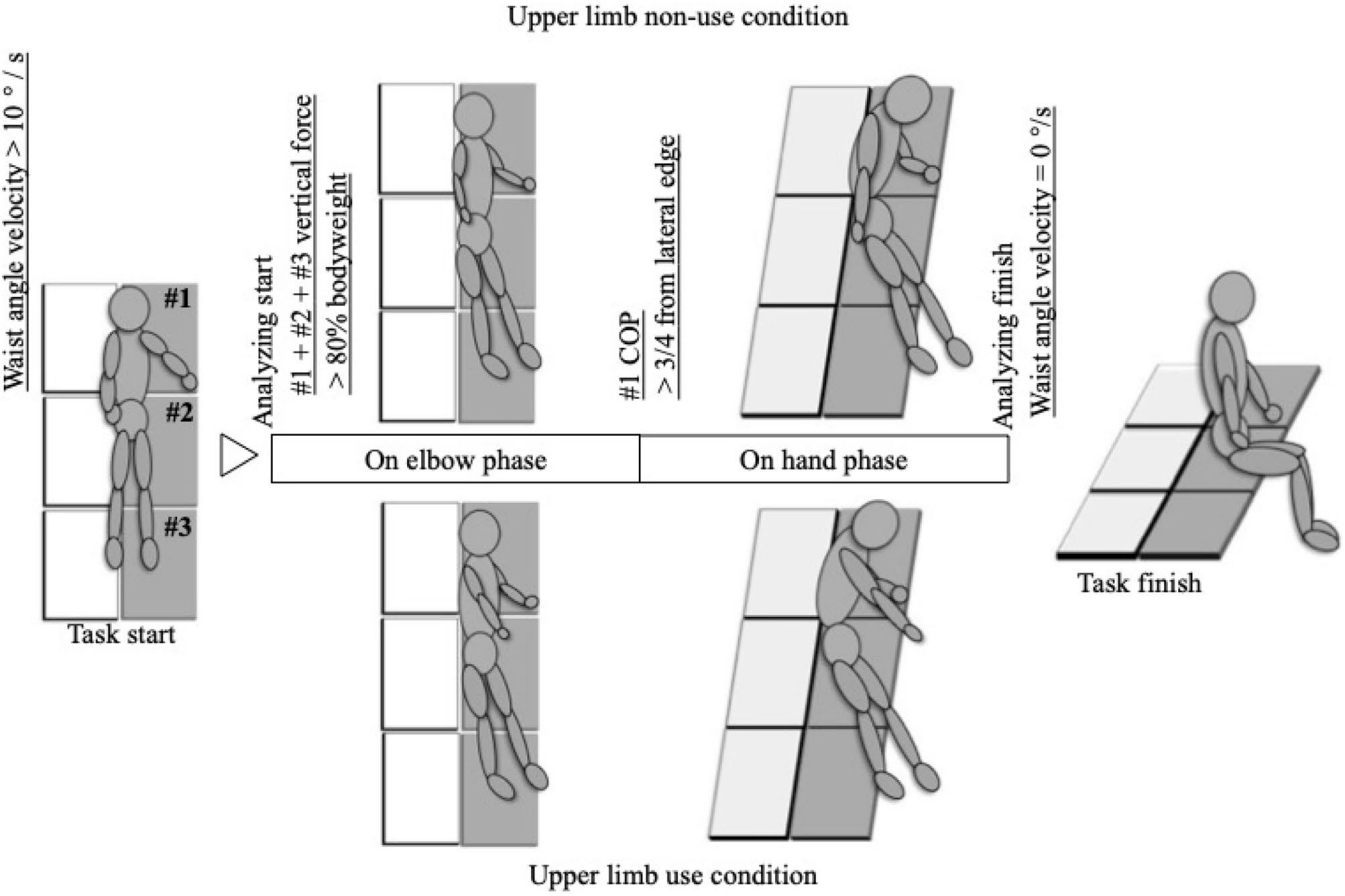
Task conditions and phase definitions. The task conditions were as follows: (1) upper limb use-condition: method of rising while raising right upper limb to the rising side; and (2) upper limb non-use-condition: method of rising while not raising the upper limb and keeping right upper limb on the right side of the pelvis from the starting posture. As the participant rises, they support their body with their left elbow (on-elbow), then with their left hand (on-hand), and finally come to rest in the sitting posture without the support of upper limbs as the final limb position. The participants were not allowed to raise their lower limbs and swing them down due to recoil or change left upper limb positions before getting up. The phases are as follows: (1) supine position (start of movement): the waist flexion angular velocity is at least 10°/s; (2) half-side-lying position: the total z-component of the three force plates on the rising side exceeds 80% of the body weight, after which the on-elbow position is reached; (3) on-hand position: when the CoP of force plate #1 that touches the upper limb moves outward by a quarter of the total distance from the outer side; and (4) sitting (end of movement): when the waist flexion angular velocity becomes 0°/s.

The movement involved in rising from the bed is an important action for individuals recovering from the bedridden state. People who take longer to perform this action have been known to have poorly balanced activities of daily life^1–3^. Individuals often adopt movement patterns such as pushing against the bed with the hand on their non-rising side or endure an intermediary posture using both elbows against the bed to facilitate the waist flexion motion, where the waist is flexed until it reaches the half-side-lying posture and the individual can lean on their elbow on the rising side^4,5^. Once through the long-sitting, this movement becomes discontinuous and takes time^2^. To ensure continuous waist flexion and body rotation, an individual can adopt the method of raising the upper limb of the non-rising side in advance to reach the rising side, and then rising diagonally toward a seating surface, thus assuming the edge-sitting via on-elbow and on-hand postures, wherein the individual leans on the hand of the rising side (Fig. 1).

It has already been shown that approximately 20–40% of elderly people cannot get up without the use of the upper limbs on the non-rising side^2^; for example, rehabilitation for patients with post-stroke hemiplegia with motor paralysis in the upper limb and lower limb of one side often focuses on raising the non-rising upper limb with motion guidance and caregiving^6^. In other words, the contribution of the upper limb of the non-rising side to the motion of getting up is established. However, the mechanical effects of upper limb use in this regard remain unclear. Despite the importance of rising movements, only a few studies have analyzed them via motion analyses. To our knowledge, no recent studies have investigated rising motions through motion analysis. This may be because there are numerous rising movements with different movement patterns; therefore, it is difficult to define them as a particular movement task^7^. The variety of movement patterns has been confirmed in healthy young^4^ and middle-aged individuals^5^. Moreover, three-dimensional (3D) motion analysis performed by attaching reflective markers to the entire body is unsuitable for this movement because an individual is in the recumbent. Lumbar flexion movements are 3D movements that occur via interaction with the hip joint^8^. Furthermore, observing the movement of the center of mass is important for assessing transitions in postures, such as those involved while getting up (from sitting to standing)^9^. Hence, there is a need for motion capture in the analysis of rising movements.

Furthermore, it has been stated that during the posture-changing motion required for changing from sitting to standing, which opposes gravity, the appropriate floor reaction force generated by the synchronization of the lower limbs and waist contributes to anti-gravity activity^10,11^. Therefore, in the analysis of rising movements, it is essential to use the timing of the waist and lower limb movements, along with the floor reaction force. The objective of this study was to clarify the effect of raising the upper limb of the non-rising side on the rising movement. Therefore, participants were provided two constraint conditions of either raising the upper limb of the non-rising side or keeping the upper limbs in contact with the pelvis. Use of the upper limbs when standing up from the sitting results in a smaller ankle dorsiflexion angle, making it easier to stand up^12^. We hypothesized that individuals who are unable to use their upper limbs, which makes it difficult to get up, compensate by adjusting the timing of waist flexion. To clarify the effect of using the upper limb of the non-rising side in isolation, the movement pattern was strictly defined. Additionally, 3D motion analysis was conducted using an original model that did not require markers attached to the participants’ backs.

## Methods

### Participants

Twelve healthy young adults (five males; age: 26.0 ± 6.2 years, weight: 58.4 ± 7.4 kg, height: 166.0 ± 6.51 cm) with no physical problems and neuromuscular disease were included. The sample size was similar to previous studies of same experiment^13,14^. We confirmed that the sample size was satisfied after being estimated using G*Power 3.1 software (Franz Faul, University of Kiel, Kiel, Germany), with an effect size 0.76, a minimum power 0.80, and α = 0.05. To avoid the failure task on non-use upper limb condition and the different movement between both conditions, we recruited young adults and were practiced tasks well. We eliminated the possibility of being unable to complete the process of getting up or having significantly different methods due to upper limb non-use. All experiments were conducted after obtaining informed consent from the participants in accordance with the Declaration of Helsinki and approval from the affiliated institutional review board.

### Measurement and task

**Figure 2:**
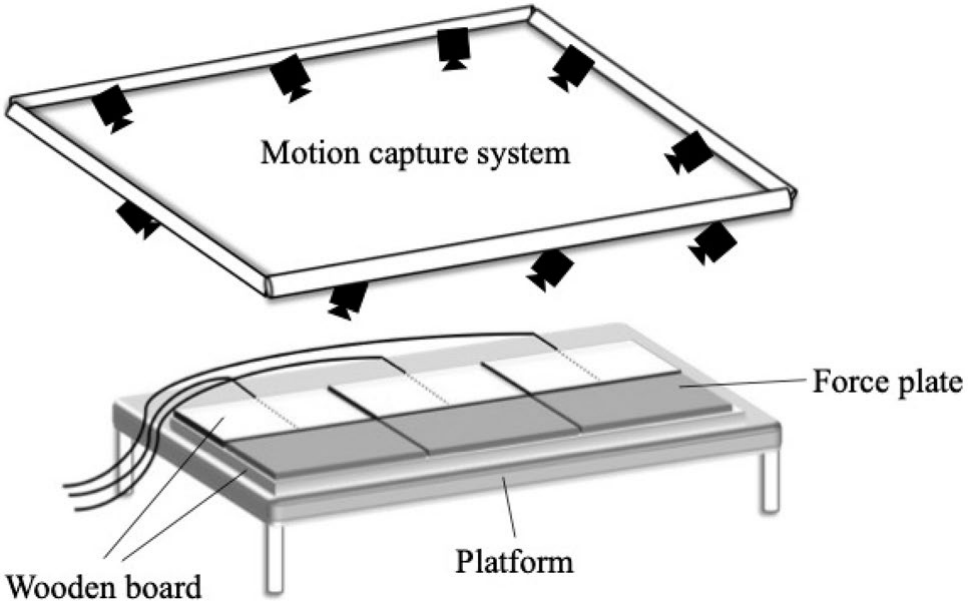
Experimental equipment. We used wooden boards that were placed on an inelastic clinical table; three force plates (1000 Hz, Kistler, 9286BA) and three wooden boards of the same size; and a Vicon 3D motion analysis device (nine cameras, 100 Hz, Vicon Motion Systems, MX T-series).

**Figure 3:**
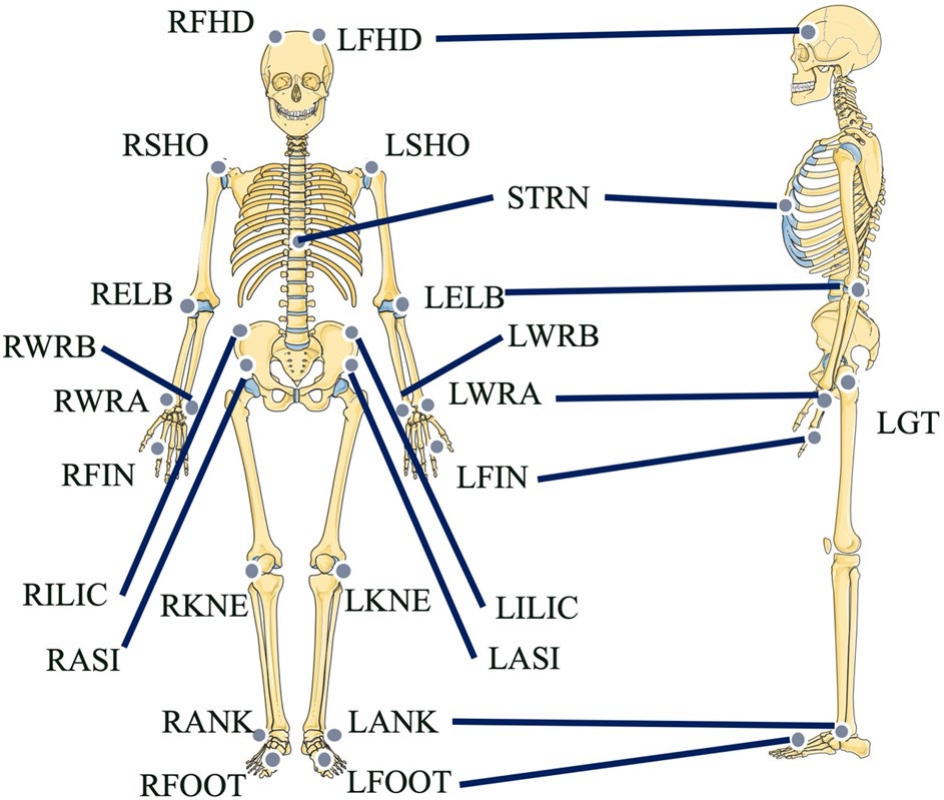
Original marker set. LFHD, RFHD: front head; STRN: xiphoid process of the sternum; LASI, RASI: anterior superior iliac spine; LILIC, RILIC: iliac crest; LGT, RGT: greater trochanter; LSHO, RSHO: acromio-clavicular joint; LELB, RELB: elbow; LWRA, RWRA: wrist bar thumb side; LWRB, RWRB: wrist lateral; LFIN, RFIN: the dorsum of the hand just below the head of the second metatarsal; LKNE, RKNE: knee on the lateral; LANK, RANK: lateral malleolus; LFOOT, RFOOT: second metatarsal head of the foot. Parts of the figure were drawn by using pictures from Servier Medical Art. Servier Medical Art by Servier is licensed under a Creative Commons Attribution 3.0 Unported License (https://creativecommons.org/licenses/by/3.0/).

A wooden board was laid on a non-elastic clinical table, after which three force plates (1000 Hz, Kistler) were placed in addition to three wooden boards of the same size as the scales (Fig. 2). The rising movement was photographed using the original marker sets (appendix, S1) shown in Fig. 3. We used a Vicon 3D motion analysis device (100 Hz, Vicon Motion Systems) equipped with nine infrared cameras.

The getting up task was from supine with left upper limb abducting to supported the body with left elbow (on-elbow), then with left hand (on-hand) and finally achieved a sitting. Two conditions are (1) upper limb use-condition, in which the participant rises while raising right upper limb to the rising side, and (2) upper limb non-use-condition, in which the participant rises while keeping right upper limb on the right side of the pelvis from the starting posture without raising it. The detailed of the task and the definition of the phase are shown in Fig. 1.

### Parameters (Fig. 4) and data analysis

**Figure 4:**
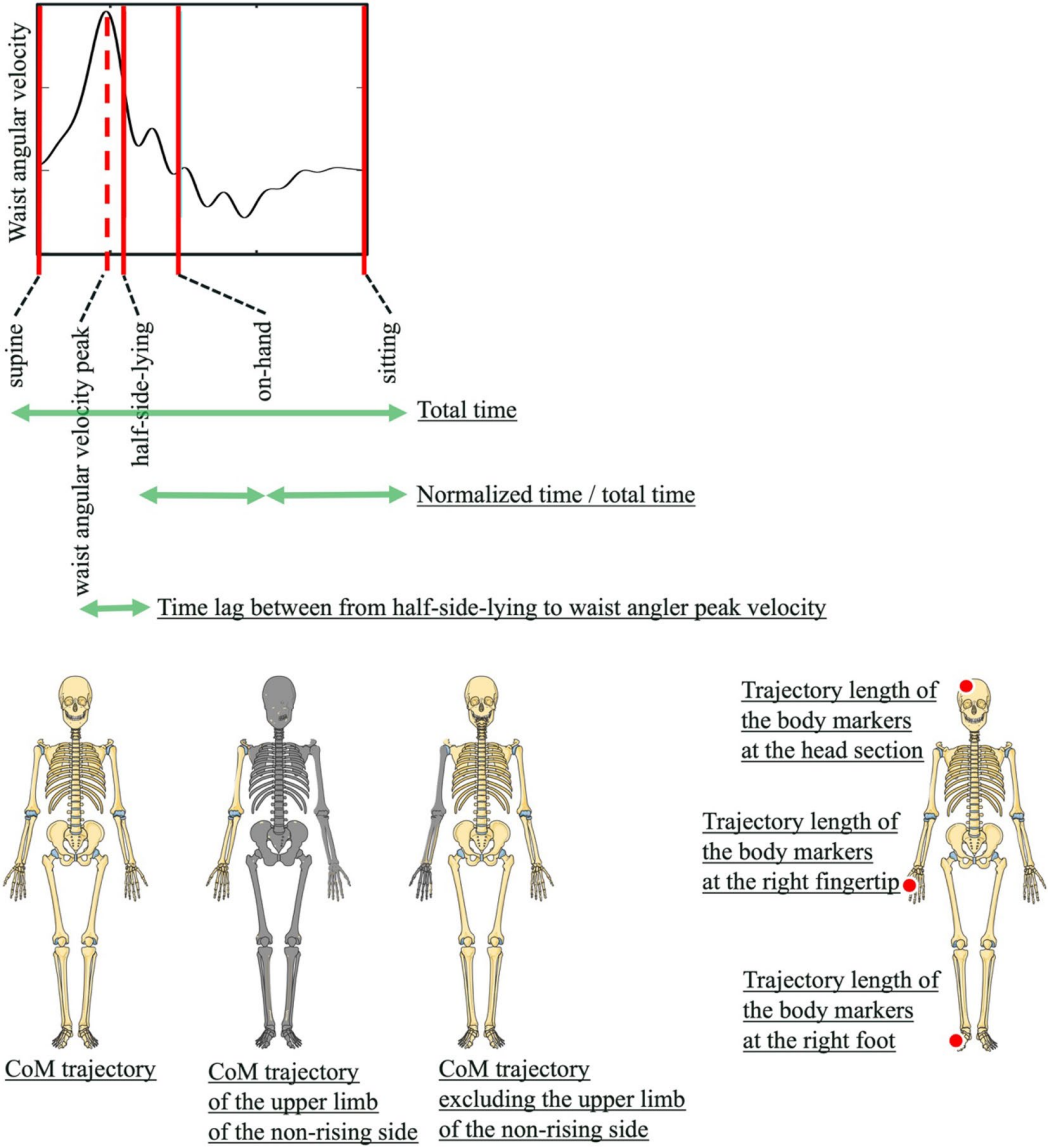
Parameters. Time parameters were total time (supine-sitting), normalized time (half-side-lying-on-hand/total time and on-hand-sitting/total time) and the lag time between the half-side-lying and the time when peak waist flexion angular velocity. Spatial parameters were trajectory length (CoM, CoM of non-rising side upper limb, CoM excluding non-rising side upper limb, right fingertip marker, right foot marker and head marker), mean waist angle, mean waist angular velocity and mean CoM momentum in horizontal and sagittal plane. Parts of the figure were drawn by using pictures from Servier Medical Art. Servier Medical Art by Servier is licensed under a Creative Commons Attribution 3.0 Unported License (https://creativecommons.org/licenses/by/3.0/).

The following time variables were calculated: (1) all phases and the time required for switching from a supine to sitting, (2) time required for reaching the on-hand from the half-side-lying, and (3) time required for reaching the sitting from the on-hand. The time required for getting up represents the degree of difficulty of the task. Additionally, all phases were 100% normalized and the lag between the half-side-lying time, during which the participant would be in a half-side-lying, and the time when peak waist flexion angular velocity would be achieved was extracted. The upper limbs work to minimize the perturbations that generate momentum^15^; thus, we extracted the duration of half-side-lying, which increases the degree of freedom, and the timing lag of the waist flexion movement, which is the maximum perturbation of the amount of movement in the rising motion.

The following spatial parameters were calculated for each of the aforementioned phases (1)–(3): the total trajectory length of the markers (right head section, right foot, right hand) in 3D, total trajectory length of centre-of-mass (CoM) in 3D (whole-body CoM, CoM of upper limb of the non-rising side, CoM excluding upper limb of the non-rising side), average waist flexion angle, average waist flexion angular velocity, and average body movement momentum on the horizontal and sagittal planes (CoM velocity/body weight). Body momentum is a variable that changes depending on upper limb use/non-use during movement. Notably, the presence or absence of upper limbs has been reported to influence angular momentum^16^. Furthermore, slight changes in arm swing affect vertical angular momentum and floor reaction force^17^.

The kinematics and dynamics data were aligned to 100 Hz. Low-pass filters of 15 and 50 Hz were applied to the kinematics and dynamics data, respectively, with a 4th-order Butterworth filter^18^. The Lilliefors test was performed for confirmation the normality of all parameters. A paired t-test and Cohen’s d were performed for comparison between the conditions within the participant. The level of significance was set at 5% (p < 0.05). MATLAB software (MathWorks) was used for all analyses.

## Results (Table 1)

**Table 1:** The phases are described as follows: supine–sitting: the entire phase from start of movement (the waist flexion angular velocity reached at least 10°/s) to end of movement (the waist flexion angular velocity became 0°/s); half-side-lying–on-hand: the total z-component of the three force plates on the rising side exceeded 80% of the body weight, thus arriving at the on-elbow state, until the center of contact pressure (CoP) of force plate #1 that touched the upper limb moved outward by a quarter of the total distance from the outer side; and on-hand–sitting: from the previously-mentioned on-hand to sitting at the end of the movement.

Parameter	Phase	With arm	Without arm	*p*-value
Total time (ms)	Supine—sitting	380 ± 58	377 ± 64	0.85
Normalized time/total time (%)	Half-side-lying—on-hand	0.12 ± 0.05	0.15 ± 0.07	< 0.01*
	On-hand—sitting	0.47 ± 0.14	0.47 ± 0.08	0.92
Time lag between from half-side-lying to waist angler peak velocity (ms)		90 ± 74	59 ± 42	< 0.05
CoM trajectory (mm)	Supine—sitting	659 ± 56	660 ± 75	0.99
	Half-side-lying—on-hand	124 ± 59	158 ± 71	< 0.01**
	On-hand—sitting	271 ± 86	269 ± 57	0.92
CoM trajectory of the upper limb of the non-rising side (mm)	Supine—sitting	58 ± 6	54 ± 8	0.20
	Half-side-lying—on-hand	10 ± 5	15 ± 7	< 0.01**
	On-hand—sitting	22 ± 8	24 ± 5	0.53
CoM trajectory excluding the upper limb of the non-rising side (mm)	Supine—sitting	608 ± 51	613 ± 71	0.68
	Half-side-lying—on-hand	115 ± 55	145 ± 65	< 0.01**
	On-hand—sitting	252 ± 80	248 ± 54	0.84
Trajectory length of the body markers at the right fingertip (mm)	Supine—sitting	1639 ± 362	837 ± 241	< 0.01**
	Half-side-lying—on-hand	229 ± 128	218 ± 97	0.68
	On-hand—sitting	529 ± 263	372 ± 138	0.09
Trajectory length of the body markers at the right foot (mm)	Supine—sitting	1605 ± 194	1630 ± 248	0.59
	Half-side-lying—on-hand	403 ± 189	499 ± 211	0.07
	On-hand—sitting	609 ± 313	514 ± 151	0.20
Trajectory length of the body markers at the head section (mm)	Supine—sitting	1551 ± 109	1547 ± 163	0.91
	Half-side-lying—on-hand	232 ± 123	306 ± 161	< 0.01**
	On-hand—sitting	641 ± 209	643 ± 165	0.96
Mean waist flexion angle (°)	Supine—sitting	41.41 ± 9.43	42.92 ± 9.48	0.45
	Half-side-lying—on-hand	55.78 ± 17.23	58.44 ± 13.31	0.58
	On-hand—sitting	48.20 ± 16.43	55.00 ± 15.92	0.05
Mean waist flexion angular velocity (°/ s)	Supine—sitting	10.61 ± 4.27	13.21 ± 7.45	0.09
	Half-side-lying—on-hand	14.84 ± 20.89	19.73 ± 16.55	0.33
	On-hand—sitting	− 12.44 ± 9.52	− 8.73 ± 10.82	0.32
CoM momentum in horizontal plane (mm/s * kg)	Supine—sitting	353 ± 118	362 ± 115	0.80
	Half-side-lying—on-hand	122 ± 93	175 ± 116	< 0.05*
	On-hand—sitting	188 ± 105	203 ± 76	0.46
CoM momentum in sagittal plane (mm/s * kg)	Supine—sitting	418 ± 110	428 ± 129	0.79
	Half-side-lying—on-hand	257 ± 136	268 ± 137	0.65
	On-hand—sitting	271 ± 143	246 ± 62	0.47

*p < 0.05, **p < 0.01.

Regarding the temporal factors, no significant differences were found between the conditions for the time required for getting up across all phases (p = 0.85, d = 0.19) and for switching from the on-hand to sitting (p = 0.92, d = 0.10), but significant differences were found in the time required to switch from the half-side-lying to on-hand (p = 0.01, d = − 3.11). The lag between the half-side-lying and peak waist flexion angular velocity was significantly lesser in the upper limb non-use-condition (p < 0.05, d = 2.58).

For the spatial factors, significant differences were observed for the trajectory length of the body markers at the right fingertip in all phases (p < 0.01, d = 5.64), but no significant differences were found for the head section (p = 0.91, d = 0.11) and right foot (p = 0.59, d = − 0.54). No significant differences were found for the total trajectory length of the whole-body CoM across all phases (p = 0.99, d = − 0.01) and from the on-hand to sitting (p = 0.92, d = 0.10), but significant differences were found in that from the half-side-lying to on-hand (p < 0.01, d = − 3.30). No significant differences were observed for the total trajectory length of the CoM of the upper limb of the non-rising side across all phases (p = 0.20, d = 1.34) and from the on-hand to sitting (p = 0.53, d = − 0.64), but significant differences were found in that from the half-side-lying to on-hand (p < 0.01, d = − 5.33). No significant differences were found for the total trajectory length of the CoM excluding the upper limb of the non-rising side across all phases (p = 0.68, d = − 0.42) and from the on-hand to sitting (p = 0.84, d = 0.20), but significant differences were found in that from the half-side-lying to on-hand (p < 0.01, d = − 3.86). No significant differences were found for the average waist flexion angle across all phases (p = 0.45, d = − 0.77), from the half-side-lying to on-hand (p = 0.58, d = − 0.56), and from the on-hand to sitting (p = 0.05, d = − 2.11). No significant differences were found for the average waist flexion angular velocity across all phases (p = 0.09, d = − 1.85) from the half-side-lying to on-hand (p = 0.33, d = − 1.01), and from the on-hand to sitting (p = 0.32, d = − 1.02). No significant differences were found for the physical momentum of the sagittal plane across all phases (p = 0.79, d = − 0.27), from the half-side-lying to on-hand (p = 0.65, d = − 0.45), and from the on-hand to sitting (p = 0.47, d = 0.74). No significant differences were found in the horizontal plane across all phases (p = 0.80, d = − 0.25) and from the on-hand to sitting (p = 0.46, d = − 0.75), but significant differences were found in that from the half-side-lying to on-hand (p < 0.05, d = − 2.21).

## Discussion

The objective of this study was to elucidate the effect of raising the upper limb of the non-rising side before waist flexion on the rising movement. Comparisons were made between the condition for raising the upper limb of the non-rising side and that for not using the upper limb. The results showed that the movement distance of the CoM from the half-side-lying to on-hand and the horizontal body momentum during the same period increased; additionally, the lag between the half-side-lying and peak waist flexion angular velocity decreased in the upper limb non-use-condition.

No differences were observed between the two conditions regarding the time required to get up, waist flexion angle, and CoP trajectory. However, significant differences were found in the trajectory of the marker distal to the body only in the upper limb of the non-rising side. Therefore, it is unlikely that the patterns of movements involved in getting up differed significantly in both conditions, except with regard to the upper limb of the non-rising side. Asymmetric rising movements, wherein the contact surface of the body is secured via the side-lying, is commonly observed in children and older adults^19^. Contrastingly, adults select symmetrical movement patterns^19^. Asymmetrical movement patterns with few body contact surfaces without assuming a complete side-lying, such as the movement task in this study, requires a certain degree of physical fitness. However, these movements are considered continuous and smooth; they require minimal body movement and have been proven to be effective as a form of movement^20^. Differences were observed in the phase between the half-side-lying and on-hand, where the participant supported their body with their palm, depending on whether the upper limb was used in this movement pattern. In both conditions, the upper limb of the non-rising side was not supposed to come in contact with the bed; therefore, the phase between the half-side-lying and on-hand was unstable and highly flexible, with only the buttocks and upper limb on the rising side in contact with the bed. In this task, the body is carried diagonally around the contact surface between the buttocks and upper limb of the rising side while bending the waist with lateral bending and rotation so that the movements are not scattered^21^. The waist flexion angle and horizontal-direction momentum were large in the condition where the upper limb of the non-rising side could not be used. These results showed that the waist flexed instantly and had horizontal momentum^22,23^. The lag between the start of the side-lying and peak waist flexion angular velocity time was also small. It is important that the CoM speed and timing of leaving the bed match even in transition operations such as sitting to standing^24^. These results showed that participants implemented a strategy in the upper limb non-use-condition to match the peak waist flexion angular velocity with the starting point of the half-side-lying, in which instability became prominent. Research on abdominal movement (getting up) have also shown that the on/off activity of the waist and hip flexors is consistent^14^. Moreover, it can be inferred from the current findings that temporal synchronization was important for anti-gravity activity, where the waist and lower limbs have a high degree of freedom. Practically, the importance of synchronizing the timing of the lower limbs and waist is stated in a similar posture-changing motion of sitting to standing^10^. There were no differences between conditions across all phases for the waist flexion angle, and the participants did not make any movement to significantly flex the waist itself in response to upper limb non-use. This was supported by the results regarding momentum in the sagittal plane, where no differences were observed. While considering past findings on abdominal movement (sit-up), it can be confirmed that minimal changes were observed in waist flexion when abdominal muscle movement was inhibited^13^. Moreover, it is possible that the extent of waist flexion during the rising movement is more limited than the range of motion. Thus, under the present upper limb non-use-condition, it can be suggested that the rise was achieved by synchronizing the peak of the waist flexion angular velocity with the starting point of the time where instability increased (half-side-lying), and then increasing the horizontal momentum. Furthermore, in support of our hypothesis, it has been shown that the contribution of the non-rising upper limb to the rising motion minimizes momentum-producing perturbations^15^ and helps the rising action with a small amount of angular momentum^16,17^.

No differences were observed in the waist flexion angle in the upper limb non-use-condition, but the degree of movement in the CoM in 3D increased. The degree of movement in the CoM excluding the upper limb of the non-rising side increased as well. Interestingly, the amount of movement in the CoM of the upper limb of the non-rising side was also large in the upper limb non-use-condition. This implies that the amount of movement in the CoM of the upper limb was suppressed in the upper limb use-condition, where the upper limb was raised. We speculate that this was caused by the horizontal movement of the upper limb of the non-rising side in the rising direction in advance during the upper limb use-condition, where the limb stayed in the raised position until the waist was raised. The mass ratio of the upper limb on the non-rising side relative to the CoM was approximately 5%, but it is presumed that approximately 37% was used in combination with both lower limbs (mass ratio approximately 32%), which contributed as a weight to move the waist to the rising side while bending it.

Based on these results, instructions to raise the upper limb on the non-rising side may be advantageous for individuals who experience difficulty in getting up. The rationale behind this is that raising the upper limb prior to waist flexion will help support their mass in combination with the lower limbs in order to raise the upper body. Furthermore, this action helps balance the unstable side-lying where only the buttocks and upper limb on the rising side are in contact with the bed. This study outlines how to guide the raising of the upper limb on the non-rising side in this rehabilitation strategy.

The results of this study showed that the compensatory movement caused by the upper limb non-use-condition caused an increase in the momentum of the body in the horizontal plane, rather than in the sagittal plane. Therefore, the elevation of the non-rising side was responsible for the participants smoothly moving their body in the horizontal direction. Moreover, the asymmetrical rising movement in the diagonal direction used in this study was proven to be a characteristic movement wherein the horizontal body movement constituted the main movement. The movements of waist flexion and rotation are often the primary focus while assisting and instructing patients under nursing care or rehabilitation with respect to the rising movement. However, the current findings indicate the need to focus on the body movement in the horizontal direction when assisting and instructing individuals regarding the rising movement. A certain degree of waist flexion, rotation strength, and range of motion are required for this movement, but strengthening them may not necessarily contribute to successfully being able to get up. It is important to have the balance required to rotate the body by 90° and correctly time the utilization of the body’s momentum while maintaining the waist flexion position. Moreover, this analysis indicated that raising and supporting the upper limb may contribute to decreasing the difficulty of the rising movement. Therefore, it is necessary to actively encourage the use of upper limbs in patients under rehabilitation and long-term care to ensure that they can independently achieve this movement.

This study had some limitations. First, the sample size was set according to those used in previous studies. Second, we included only healthy young adults; thus, generalization to other participants is limited. Additionally, we applied many constraints to the task movement in order to emphasize the effect of raising only the upper limbs. The limitation was that this was not the most comfortable or suitable way for the participants to get up. However, with sufficient practice, they got used to the experiment. Additionally, the model that was developed in this experiment may have reduced accuracy regarding the measurement of deep waist flexion angles. It has been confirmed that none of the participants in this study conducted a flexion movement of ≥ 90°. In future studies, we plan to include more participants in this study so that these results can be validated against a larger dataset. Future experiments that target older adults who are at high risk of being bedridden may yield methods and results that are unique to those groups.

## Supplementary Information

Supplementary Information.

## Data Availability

The data set from the study are held securely in coded form by the corresponding author, K. H. The data underlying this article will be shared on reasonable request to the corresponding authors after granting prespecified criteria for confidential access.
