# Supplementary material for: Role of raising the upper limb of the non-rising side when performing rising movements from bed
Source: Sci Rep. 2023 Jul 16;13:11475. doi: 10.1038/s41598-023-38779-2 (PMC10350449; doi:10.1038/s41598-023-38779-2)
Supplement: Supplementary file 1 — Supplementary Information. [file 41598_2023_38779_MOESM1_ESM.docx]

**SUPPLEMETARY INFORMATION**

*Original Model of Motion Analysis- Detailed Methods*

In this experiment, we estimated the CoM and waist angle with the original model using our own marker set. The waist angle was calculated as the relative angle between the pelvis and thoracic segment. First, the coordinate systems for the two segments were defined with respect to a global coordinate system. The directions of the X, Y, and Z axes in the pelvic coordinate system were defined as follows: (1) X axis, vector passing through the left/upper right anterior iliac spine; (2) pseudo Z axis, vector passing through the apex of the right iliac crest and center of the right hip joint; (3) Y axis, cross product of X axis and pseudo Z axis; and (4) Z axis, cross product of X and Y axes. The directions of the X, Y, and Z axes in the thoracic coordinate system were defined as follows: (1) X axis, vector passing through left/right acromial extremities; (2) pseudo Z axis, vector passing through the midpoint of left/right acromial extremities and sternal xiphoid process; (3) Y axis, cross product of X axis and pseudo Z axis; and (4) Z axis, cross product of X and Y axes. The vectors in the directions of the aforementioned X, Y, and Z axes were converted into unit vectors by dividing them with their respective vector lengths (orthonormal basis). The center of the hip joint was treated as the 1/3^rd^ point from the center of the greater trochanter on the line connecting the center of the great trochanter and anterior superior iliac spine.

Next, the pelvis-thoracic coordinate transformation matrix, *RTM_PtoT_*, between the coordinate systems for the two segments was calculated.

*RTM_PtoT_ = DCM_T_・(DCM_P_)^T^*

Here, *DCM_P_* and *DCM_T_* represent the pelvic and thoracic coordinate system matrices, respectively. Additionally, the superscript *^T^* refers to a transposed matrix.

Finally, this pelvis-thoracic coordinate transformation matrix was used to calculate the waist angle by XYZ Euler angle-based posture expression.

*Original Model of Motion Analysis- Accuracy Verification*

We conducted the verification of the three-way motion of the waist in advance in one case. To this end, we affixed markers for both the original model and the plug-in-gait full body AI model on one healthy young adult. The waist flexion, extension, and rotation movements were performed individually, and the mutual correlation coefficient and root-mean-square error between the CoM and waist angles in each movement direction are included in Table. The accuracy of the original model was confirmed because the original and AI models had a high correlation and low root-mean-square error.

| parameter | | correlation foefficient | RMS difference |
| --- | --- | --- | --- |
| Waist Angle | Flexion angle | r = 0.97 | 9.58 ° |
|  | Side bending angle | r = 0.99 | 2.24 ° |
|  | Rotation anlge | r = 0.99 | 3.83 ° |
|  |  |  |  |
| Centre of mass | Saggital | r = 0.97 | 13.84 mm |
|  | Frontal | r = 0.97 | 15.70 mm |
|  | Horizontal | r = 0.97 | 9.58 mm |

**S1:** Accuracy of the original model. Comparison results of the original model and plug-in-gait full body AI model.
